# Chromoblastomycosis in Western Thailand

**DOI:** 10.4269/ajtmh.2010.10-0210

**Published:** 2010-09

**Authors:** Philip McDaniel, Douglas S. Walsh

**Affiliations:** Kwai River Christian Hospital, Sangklaburi (Kanchanaburi Province), Thailand; Department of Immunology and Medicine, United States Army Medical Component, Armed Forces Research Institute of Medical Sciences (AFRIMS), Bangkok, Thailand

**Figure 1.**
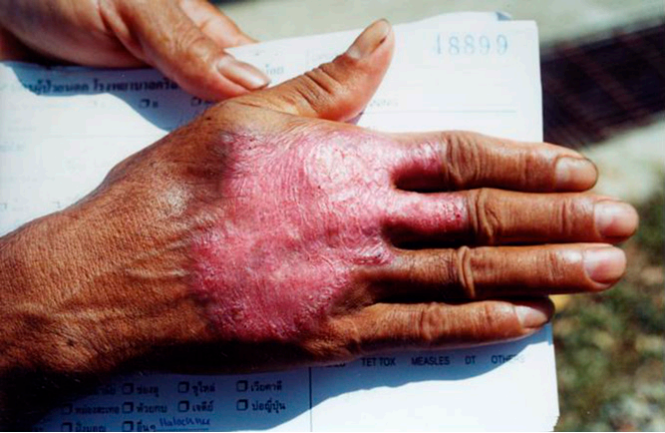
Well-demarcated plaque on the dorsal surface of hand. This figure appears in color at www.ajtmh.org.

**Figure 2.**
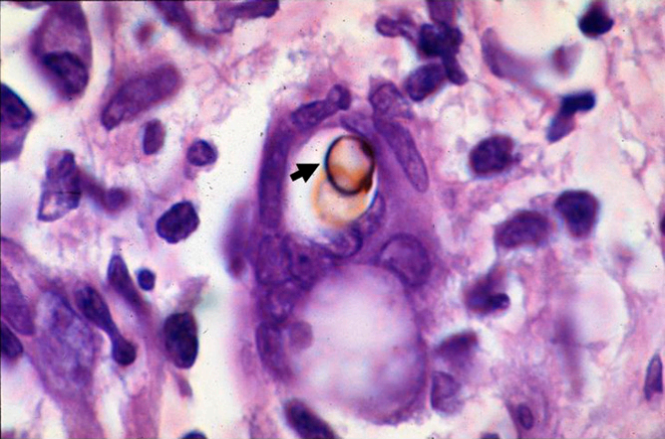
Histology shows mononuclear cell infiltrate and a dark-brown, round sclerotic body resembling a “copper penny” (arrow), consistent with chromoblastomycosis (hematoxylin-eosin stain; 1,000×). This figure appears in color at www.ajtmh.org.

A man living in rural western Thailand presented with a well-demarcated pinkish plaque on the dorsal surface of the right hand, extending to several fingers (Figure 1). Mild scale was present. The lesion was not pruritic or tender, and there was no sporotrichoid lymphadenopathy. The differential diagnosis included cutaneous deep fungal and atypical mycobacterial infections. A punch biopsy showed a mononuclear dermal infiltrate with multinucleated giant cells and scattered dark-brown, round sclerotic bodies resembling “copper pennies” (Figure 2), features consistent with chromoblastomycosis, a cutaneous deep fungal infection. Oral terbinafine (anti-fungal sterol inhibitor) was administered at 250 mg two times daily for 2 weeks and then, 250 mg daily for 14 weeks, with progressive resolution.

Chromoblastomycosis, caused by a saprophytic pigmented (dematiaceous) fungus, occurs in many tropical areas, including Thailand.^1^ It may be acquired by traumatic implantation, such as a wood splinter contaminated with fungal elements. Regional lymphatic damage and malignant transformation may occur. Treatment options include oral anti-fungal medications and physical methods, the former often requiring lengthy courses, and responses vary.^1^,^2^ Here, we speculate that terbinafine dosed at 500 mg daily for the first 2 weeks, a less commonly prescribed higher daily dose, may have been beneficial.
